# AGHE Faculty Award Presentations and Lectures

**DOI:** 10.1093/geroni/igad104.1212

**Published:** 2023-12-21

**Authors:** Dana Bradley

## Abstract

The AGHE Faculty Award Presentations and Lectures will feature an address by the 2023 recipient Lisa Borrero, PhD, FAGHE of the University of Indianapolis. The Distinguished Faculty Award recognizes persons whose teaching stands out as exemplary, innovative, of impact, or any combination thereof. The Rising Star Early-Career Faculty Award Lecture will feature an address by 2023 co-recipient Nasreen Sadeq, PhD of the University of Southern Florida. Co-recipient Berenice Benayoun, PhD, of the University Southern California will present their lecture at a later date. The Rising Star Early-Career Faculty Award acknowledges new faculty whose teaching and leadership stand out as influential and innovative.

